# Reconstruction of a Genome Scale Metabolic Model of the polyhydroxybutyrate producing methanotroph *Methylocystis parvus* OBBP

**DOI:** 10.1186/s12934-019-1154-5

**Published:** 2019-06-07

**Authors:** Sergio Bordel, Antonia Rojas, Raúl Muñoz

**Affiliations:** 1 Departamento de Ingeniería Química y Tecnología del medio ambiente, Escuela de Ingenierías Industriales, Universidad de Valladolid, Valladolid, Spain; 2 Institute of Sustainable Processes, Universidad de Valladolid, Valladolid, Spain; 3 Biopolis S.L., Parc Cientific Universitat de Valencia, Paterna, Spain

**Keywords:** Genome-scale metabolic models, Metabolism, Methanotrophs, Methylocystis

## Abstract

**Background:**

*Methylocystis parvus* is a type II methanotroph characterized by its high specific methane degradation rate (compared to other methanotrophs of the same family) and its ability to accumulate up to 50% of its biomass in the form of poly-3-hydroxybutyrate (PHB) under nitrogen limiting conditions. This makes it a very promising cell factory.

**Results:**

This article reports the first Genome Scale Metabolic Model of *M. parvus* OBBP. The model is compared to Genome Scale Metabolic Models of the closely related methanotrophs *Methylocystis hirsuta* and *Methylocystis* sp. SC2. Using the reconstructed model, it was possible to predict the biomass yield of *M. parvus* on methane. The prediction was consistent with the observed experimental yield, under the assumption of the so called “redox arm mechanism” for methane oxidation. The co-consumption of stored PHB and methane was also modeled, leading to accurate predictions of biomass yields and oxygen consumption rates and revealing an anaplerotic role of PHB degradation. Finally, the model revealed that anoxic PHB consumption has to be coupled to denitrification, as no fermentation of PHB is allowed by the reconstructed metabolic model.

**Conclusions:**

The “redox arm” mechanism appears to be a general characteristic of type II methanotrophs, versus type I methanotrophs that use the “direct coupling” mechanism. The co-consumption of stored PHB and methane was predicted to play an anaplerotic role replenishing the serine cycle with glyoxylate and the TCA cycle with succinyl-CoA, which allows the withdrawal of metabolic precursors for biosynthesis. The stored PHB can be also used as an energy source under anoxic conditions when coupled to denitrification.

**Electronic supplementary material:**

The online version of this article (10.1186/s12934-019-1154-5) contains supplementary material, which is available to authorized users.

## Background

*Methylocystis parvus* is a type II methanotroph characterized by a specific growth rate higher than other species of the genus *Methylocystis* (such as *Methylocystis hirsuta* or *Methylocystis* sp. SC2) and that, under nitrogen limiting conditions, is able to accumulate poly-3-hydroxybutyrate (PHB) up to 50% of its dry biomass.

Methane is a powerful green-house effect gas, which is currently being emitted at a rate over 70 million tonnes per year [1] in anthropogenic activities such as wastewater treatment, mining or landfilling. Methanotrophic bacteria, able to use methane as the sole energy and carbon source, have a great potential for the abatement of methane in end-of-the-pipe processes, thus contributing to mitigate climate change. Besides their potential for the attenuation of global warming, methanotrophic organisms are also promising cell factories [2] due to their ability to use a virtually free carbon source (indeed a waste compound from the anaerobic degradation of organic matter). In the context of sugar-based industrial biotechnology, the prize of the feedstock can account for up to 30% of the total production costs, which makes methane, even in the form of natural gas [3], a very competitive substrate. In order to fully exploit the potential of methane as a substrate for industrial biotechnology, it is necessary to develop genetic tools for the manipulation of methanotrophic strains as well as Genome Scale Metabolic Models (GSMMs) allowing to define metabolic engineering strategies. GSMMs and genetic engineering tools have been developed for two type I methanotrophs, namely *Methylomicrobium buryatense* and *Methylomicrobium alcaliphilum* [4, 5]. Type I methanotrophs rely on the ribulose monophosphate pathway for the assimilation of methane, which is previously oxidized to formaldehyde. In contrast to type I methanotrophs, type II methanotrophs, such as those of the genus *Methylocystis*, rely on the serine cycle for methane assimilation. Type II methanotrophs, so far, have not been used as metabolic engineering platforms, not even at a research level, even if successful gene knockouts have been reported for *Methylocystis* sp. SC2 [6]. On the other hand, strains of the genus *Methylocystis* are natural producers of PHB, a biodegradable polymer, similar to polypropylene in terms of mechanical properties, that is synthesized under nitrogen starvation conditions. *Methylocystis parvus* has been reported to accumulate up to 50% of its dry weight in the form of PHB [7], which makes it a very suitable industrial biopolymer producer. Other strains such as *M. hirsuta* can accumulate up to 45% of PHB on a dry weight basis using methane as a feedstock [8]. PHB production at an industrial scale involves an initial production of biomass under nutrient sufficient conditions, followed by a PHB accumulation step under nitrogen limiting conditions. The performance of the first step will be governed by the specific growth rate and methane consumption rate of the selected strain, while PHB accumulation will be determined by the metabolic capacity of the strain and should be carried out under operational conditions preventing PHB consumption by the cells. At this point, it should be kept in mind that PHB is used by many organisms as a way of storing carbon and reducing power [9], and it is used as an internal substrate for cell survival and proliferation under limitation of external carbon and energy sources.

In this work, the first GSMM of *M. parvus* OBBP was reconstructed [10]. This GSMM was compared to GSMMs of the two closely related strains *M. hirsuta* CSC1 [11] and *M.* sp. SC2 [12]. The reconstructed GSMMs of *M. parvus* was used to elucidate some relevant aspects of the physiology of PHB accumulating methanotrophs. The first question addressed was the mechanism of methane oxidation used by methanotrophic organisms. Methane is initially oxidized to methanol, thus consuming a molecule of oxygen. This step also requires a reduced redox co-factor carrying two electrons, which are transferred to the second oxygen atom to produce a molecule of water. There is no general agreement on the identity of this redox co-factor, and three alternative mechanisms have been proposed [13]. The so-called “redox arm mechanism” considers the redox co-factor to be ubiquinol, which is restored to its initial reduced state with NADH consumption in complex I of the respiratory chain. The “direct coupling” mechanism assumes that the co-factor involved in methane oxidation is cytochrome-*c*, which is reduced back during the oxidation of methanol to formaldehyde by the enzyme methanol dehydrogenase (MeDH). Finally, a third mechanism consists of the reduction of ubiquinone by cytochrome-*c* in the so called “uphill electron transfer” mechanism. GSMMs of type I methanotrophs [4, 5] were capable of accurately predicting the biomass yields on methane and oxygen/methane consumption ratios under the assumption of the direct coupling mechanism. The work herein conducted assessed if experimental biomass yields of *M. parvus* OBBP were consistent with any of the three hypothesized methane oxidation mechanisms.

*Methylocystis parvus* has been shown to use the stored PHB for protein synthesis in the absence of methane and in the presence of nitrogen sources [7]. However, cells do not divide in the absence of methane, likely due to regulatory mechanisms. When both methane and nitrogen sources are available, *M. parvus* consumes simultaneously PHB and methane while decreasing significantly its cell division time [7], which provides a competitive advantage to cells with stored PHB. The GSMM presented here will be used to assess the metabolic pathways involved in this phenomenon of co-consumption. In the absence of oxygen, *M. parvus* has been shown to produce acetic acid and butane-2,3-diol [14], which was interpreted as a fermentation mechanism that allows the cell to obtain maintenance ATP in the absence of oxygen. This mechanism will be tested in silico using the reconstructed model.

## Results

### Genome scale metabolic model of *M. parvus* OBBP compared to *M. hirsuta* and *M.* sp. SC2

A metabolic model of *M. parvus* OBBP was reconstructed using SEED [15] after annotation with RAST [16] and manually curated as described in “Materials and methods” section. The reconstructed model of *M. parvus* was made publicly available in SBML and tab-separated formats at https://github.com/SergioBordel/ModelsMethanotrophs. The same repository also contains the lists of essential reactions, lists of the top connected metabolites and RAST genome annotations in Excel format. The model is presented compared to GSMMs of two other species of *Methylocystis*, namely *M. hirsuta* and *M.* sp. SC2 [17]. General model statistics are represented in Table 1.

**Table 1:** General GSMMs statistics for the three strains evaluated in this study

	Genes	Reactions	Metabolites	Essential reactions
*M. parvus* OBBP	2795	1326	1399	380
*M. hirsuta*	2748	1350	1428	383
*M.* sp. SC2	2251	1449	1434	381

**Fig. 1:**
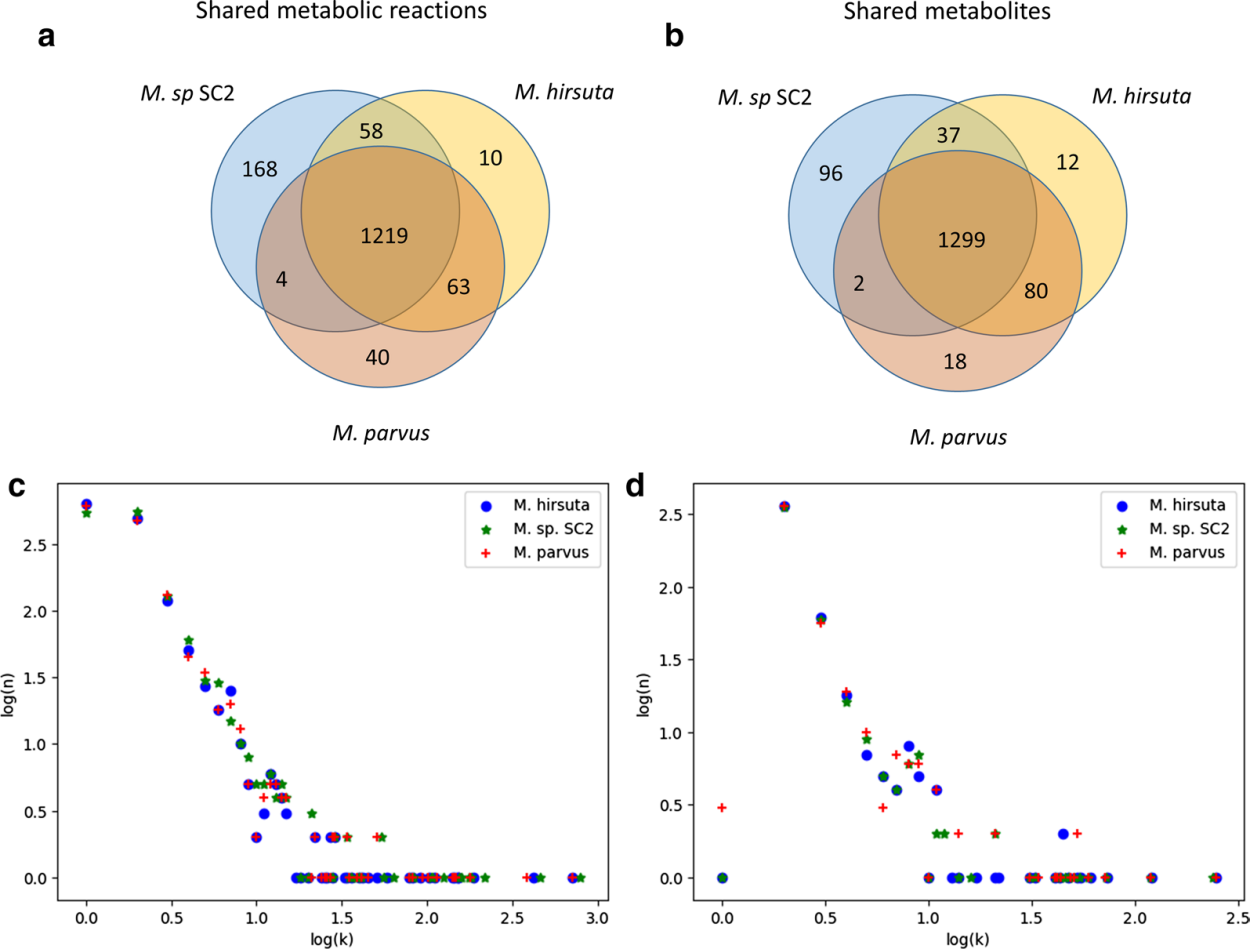
Venn diagrams representing the shared and unique reactions (**a**) and metabolites (**b**) of the GSMMs of *Methylocystis parvus OBBP*, *Methylocystis hirsuta* and *Methylocystis* sp. SC2. **c** The logarithms of the numbers (n) of metabolites participating in k reactions. The plot shows a typical scale free topology. **d** The same plot including only reactions that are active under optimal growth on methane, and their metabolites

Essential reactions are defined as those in whose absence, no biomass production from methane is possible. For *M. parvus* 28.7% of its metabolic reactions are essential for growth on methane. The overlap between the reactions and metabolites in the three different models is shown in Fig. 1a, b. From a topological point of view, the distribution of the number of reactions in which a metabolite takes place in the models is scale free, which means that the logarithm of the number of metabolites (n) participating in k reactions is linearly dependent of log(k). The slope is equal to − 2.5, which is an almost universal property of metabolic networks [18]. The most connected metabolites such as ATP, NADH and other co-factors constitute outliers, which participate in a higher number of reactions that those expected in a scale free distribution. The sub-network of reactions that are active under optimal growth on methane, also shows a scale free topology with the same slope.

Among the 40 reactions that are unique to *M. parvus*, 8 of them are catalyzed by a purine nucleoside phosphorylase (EC. 2.4.2.1). This enzyme is involved in salvage pathways that allow recovering nucleotides from the degradation of nucleic acids such as messenger RNA. This enzyme could be related to the higher specific growth rate of *M. parvus* by contributing to a faster turnover rate of messenger RNA. A similar function could be that of a nicotinamide phosphoribosyltranspherase (EC. 2.4.2.12), which is also specific to *M. parvus* and is absent in the metabolic networks of *M. hirsuta* and *M.* sp. SC2. An interesting enzyme unique to *M. parvus* is homoserine *O*-succinyltransferase (EC. 2.3.1.46), which synthesizes *O*-succinyl-l-homoserine from succinyl-CoA and l-homoserine. In contrast, the two other considered species appear to synthesize *O*-succinyl-l-homoserine from succinate and cystathionine using the reverse activity of a cystathionine-γ-synthase (EC. 1.4.1.2). Other two important metabolic genes, present uniquely in *M. parvus*, are the transporters of l-proline (l-proline/glycine betaine transporter ProP) and 3-hydroxybutanoate (d-β-hydroxybutyrate permease). The presence of these transporters could make *M. parvus* a suitable cell factory for these compounds. Indeed, this strain has been reporter to secrete butane-2,3-diol [14] under anoxic conditions, this compound could be transported outside the cell via the mentioned hydroxybutyrate permease.

The enzymes cystathionine-γ-synthase (EC. 2.5.1.48) and l-threonine 3-dehydrogenase (EC. 1.1.1.103), involved in the synthesis of amino acids, nicotinamidase (EC. 3.5.1.19), that degrades nicotinamide to niacin, adenosine deaminase (EC. 3.5.4.4) or adenosine deaminase (EC 3.5.4.4), rank among the main enzymes absent in *M. parvus* and present in both *M. hirsuta* and *M.* sp. SC1.

### Model validation, predictions of biomass yields on methane

Depending on the redox-carrier involved in methane oxidation, there are three possible mechanisms of methane oxidation (previously described). A reaction in which methane oxidation is coupled to the oxidation of cytochrome-*c* (direct coupling mechanism), has been included in the model with the identifier “pMMO1”. A second reaction, in which methane oxidation is coupled to ubiquinol-8 oxidation, has been included with the identifier “pMMO2” (note that the identifiers do not correspond to different iso-enzymes but just to possible alternative stoichiometries). The “uphill electron transfer mechanism” was herein modelled by allowing the reaction corresponding to the complex III of the respiratory chain (with identifier “rxn10113_c0”) to proceed backwards.

**Fig. 2:**
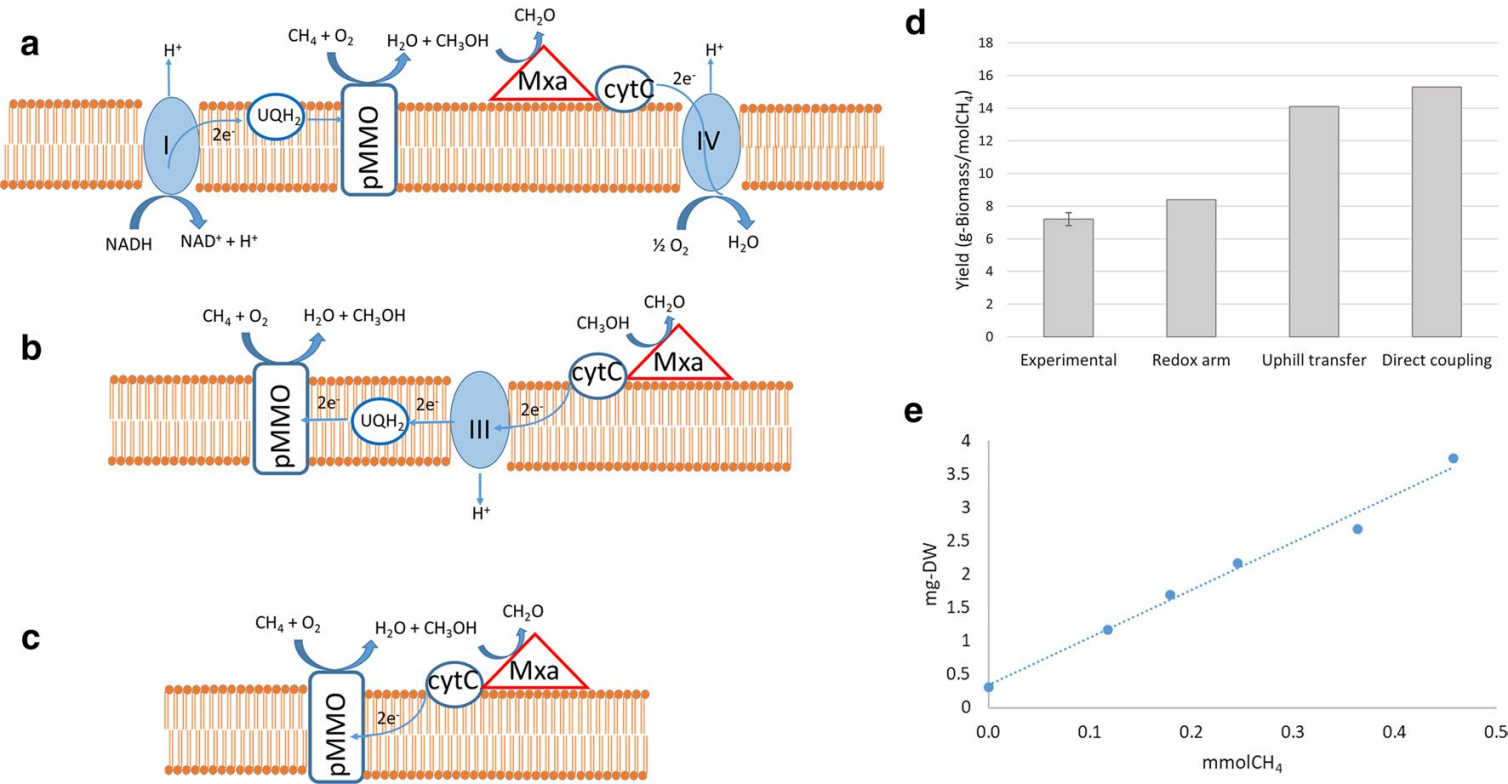
Schematic representation of the three possible methane oxidation mechanisms: “redox arm” (**a**), “uphill electron transfer” (**b**) and “direct coupling” (**c**). Comparison of the biomass yields on methane predicted by the model under each mechanism with the experimental yield observed. **d** Error bars correspond to standard deviations from triplicate experiments. *M. parvus* OBBP biomass produced as a function of methane degraded (**e**)

Experiments in closed serum flasks containing different methane concentrations in the headspace, were carried out (see “Materials and methods”). The total biomass produced versus the total methane consumed is plotted in Fig. 2e. The biomass yield on methane, obtained from the slope, was 7.2 ± 0.4 g-DW/molCH_4_. In order to obtain an estimation of the non-growth associated ATP requirements (which in the absence of methane are supplied by endogenous respiration), oxygen consumption was monitored following total methane depletion (in triplicate serum bottles), leading to a value of 0.71 ± 0.2 mmolO_2_ g-DW^−1^ h^−1^. In this context, assuming five molecules of ATP produced per oxygen molecule, the non-growth associated ATP maintenance would be 3.5 mmol ATP g-DW^−1^ h^−1^.

The doubling time of *M. parvus* OBBP was 6.45 h, which corresponded to a growth rate of 0.107 h^−1^ [7]. The specific methane uptake rate by *M. parvus* OBBP was 14.9 mmolCH_4_ g-DW^−1^ h^−1^. This experimental value, together with the estimated ATP consumption rate for non-growth associated maintenance, were set as constraints and biomass production was optimized under the assumption of each of the three possible methane oxidation mechanisms (Fig. 1a–c). The theoretical maximal yields under each assumption are represented in Fig. 1d. The experimental results confirmed that *M. parvus* OBBP uses the so-called “redox arm” mechanism, similarly to *M. hirsuta* and *M.* sp. SC2 [17]. The molar oxygen-consumption ratio of *M. parvus* was 1.5 mol O_2_ mol CH_4_^−1^ [7], which was also identical to the prediction of the model using the “redox arm” mechanism.

### Co-consumption of methane and PHB

*Methylocystis parvus* OBBP uses PHB to store carbon and energy under nitrogen limiting conditions, and once nitrogen is available anew, the stored PHB is consumed by the cells. A previous study [7] revealed that in the absence of methane and nitrogen excess, *M. parvus* OBBP consumed PHB and was able to synthesize proteins, but cells did not divide. In contrast, after 4 h of exposure to both nitrate and methane, *M. parvus* OBBP started consuming methane and PHB simultaneously and exhibited a doubling time of 4.94 h during its first duplication [7]. Subsequent duplications were slowed down due to the consumption of the stored PHB. This initial duplication time corresponded to a specific growth rate of 0.154 h^−1^, which confirmed that the presence of PHB inside the cells confers a competitive advantage versus cells without stored PHB.

During the 15 h of PHB and methane co-consumption by *M. parvus* OBBP (experiments carried out in 125 mL serum bottles with 50 mL liquid medium [7]), the PHB content of the flasks decreased from 0.16 ± 0.01 to 0.017 ± 0.002 g/L, while the non-PHB dry biomass concentration increased from 0.27 ± 0.01 to 0.69 ± 0.06 g/L. This was associated to the consumption of 1.8 ± 0.09 mmolCH_4_ and 2.6 ± 0.13 mmolO_2_ (per bottle) [7]. Considering that each bottle contained 50 mL of mineral medium, a total of 21 ± 3 mg of non-PHB dry biomass were formed in each bottle and 0.083 ± 0.007 mmol of PHB were consumed (using a molar mass equal to that of the monomer minus a water molecule).

In order to test the performance of the model, the consumed methane and PHB were set as constraints and biomass formation was optimized, leading to predictions of 21.59 mg of non-PHB dry biomass produced and 2.7 mmol of O_2_ consumed. These predictions are remarkably close to the experimental values [7].

**Fig. 3:**
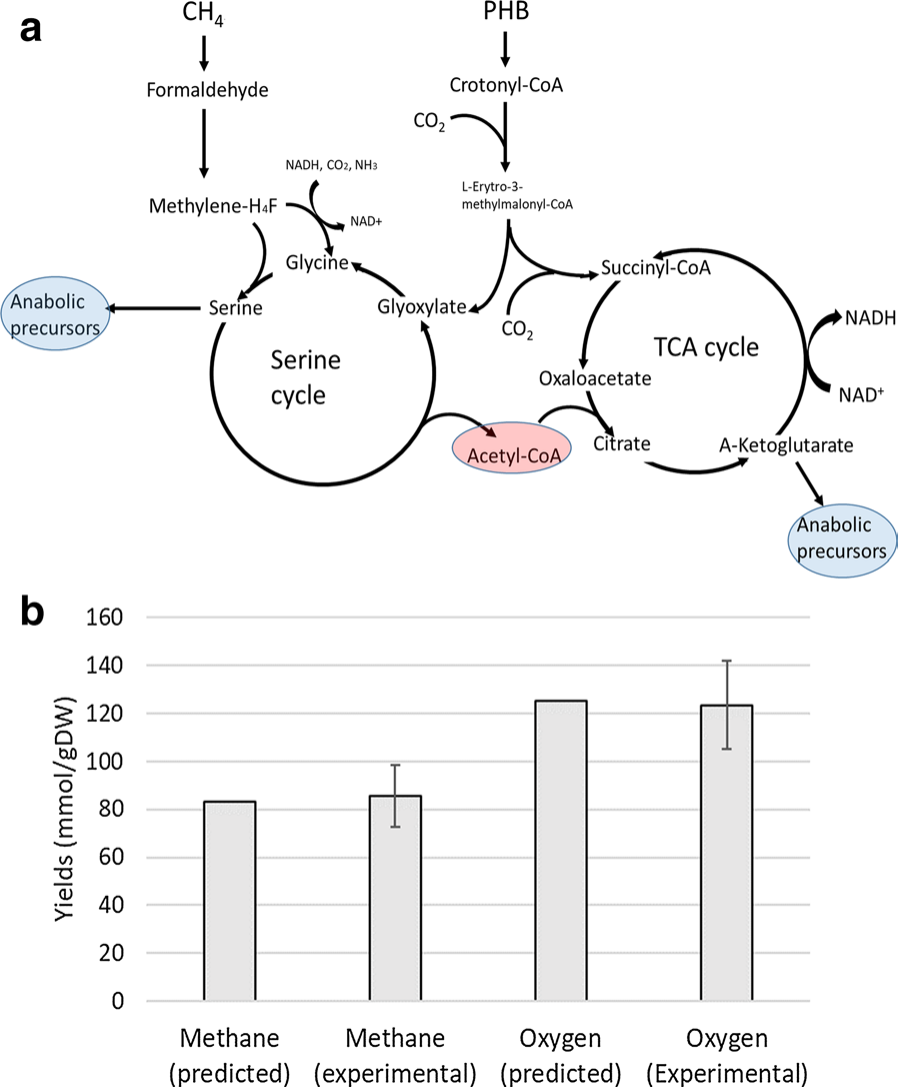
Schematic representation of the metabolic processes active during PHB and methane co-consumption (**a**). Methane and oxygen consumption per gram of produced biomass during PHB co-consumption with methane (**b**). The experimental yields of methane and oxygen consumed per unit of synthesized biomass are compared to the yields predicted by the GSMM model

The optimal distribution of metabolic fluxes involving PHB and methane co-consumption corresponds to PHB degradation to l-erytro-3-methylmalyl-CoA, which becomes dissociated into glyoxylate and propionyl-CoA (by the enzyme malyl-CoA lyase, which also breaks down malyl-CoA into glyoxylate and acetyl-CoA in the serine cycle). The glyoxylate originated from l-erytro-3-methylmalyl-CoA is incorporated into the serine cycle, while propionyl-CoA is carboxylated into succinyl-CoA and thus incorporated into the TCA cycle (Fig. 3a). PHB degradation is thus playing an anaplerotic role by supplying glyoxylate to the serine cycle and succinyl-CoA to the TCA cycle, which allows intermediates from both cycles (such as serine from the serine cycle or α-ketoglutarate from the TCA cycle) to be used as anabolic precursors to build up biomass. In the absence of external supply of metabolic intermediates, the serine cycle could only produce acetyl-CoA and the TCA cycle would consume this acetyl-CoA leading to the production of NADH, which would result in the production of energy but not in cell growth, for which biomass building blocks are necessary. Therefore, the supply of metabolic intermediates to the serine cycle and/or the TCA cycle is necessary for cell growth (this phenomenon receives the name of anaplerosis and the metabolic reactions supplying intermediates for these cycles are known as anaplerotic reactions).

During growth on methane as the sole carbon and energy source, this anaplerotic function is mainly played by glycine synthase activity, which carboxylates 5-10-methylenetetrahydrofolate (with a concomitant consumption of NADH and ammonium) to produce glycine. This glycine is incorporated into the serine cycle allowing withdrawal of serine for biosynthetic purposes. The simulations using the GSMM herein constructed predicted a rate of glycine synthesis of 1.3 mmol h^−1^ g-DW^−1^ during growth on methane, which decreased to 0.41 mmol h^−1^ g-DW^−1^ during methane-PHB co-consumption, thus confirming the lower dependence of the cells on glycine synthase due to the anaplerotic role of PHB degradation. Table 2 shows the simulated rates of methane consumption, production of acetyl-CoA by the serine cycle and glycine synthesis during growth on methane and PHB co-consumption.

**Table 2:** Rates of key metabolic processes active during PHB and methane co-consumption compared to growth on methane

	Growth on CH_4_	Co-consumption of PHB and CH_4_
*µ* (h^−1^)	0.107	0.154
Methane consumption (mmol h^−1^ g-DW^−1^)	14.9	13.2
PHB degradation (mmol h^−1^ g-DW^−1^)	0	0.608
Malyl-CoA lyase (mmol h^−1^ g-DW^−1^)	12.3	11.8
Glycine synthase (mmol h^−1^ g-DW^−1^)	1.3	0.41

At this point it should be stressed that the enzyme malyl-CoA lyase catalyzes both the lysis of malyl-CoA in the serine cycle as well as the lysis of l-erytro-3-methylmalyl-CoA, whose rate is equal to the PHB degradation rate. Therefore, it seems reasonable that the increased rate of l-erytro-3-methylmalyl-CoA lysis from zero to 0.608 mmol h^−1^ g-DW^−1^ is concomitant to a drop in the rate of malyl-CoA lysis from 12.3 to 11.8 mmol h^−1^ g-DW^−1^ due to a competitive inhibition mechanism.

### Role of stored PHB under anoxic conditions

**Fig. 4:**
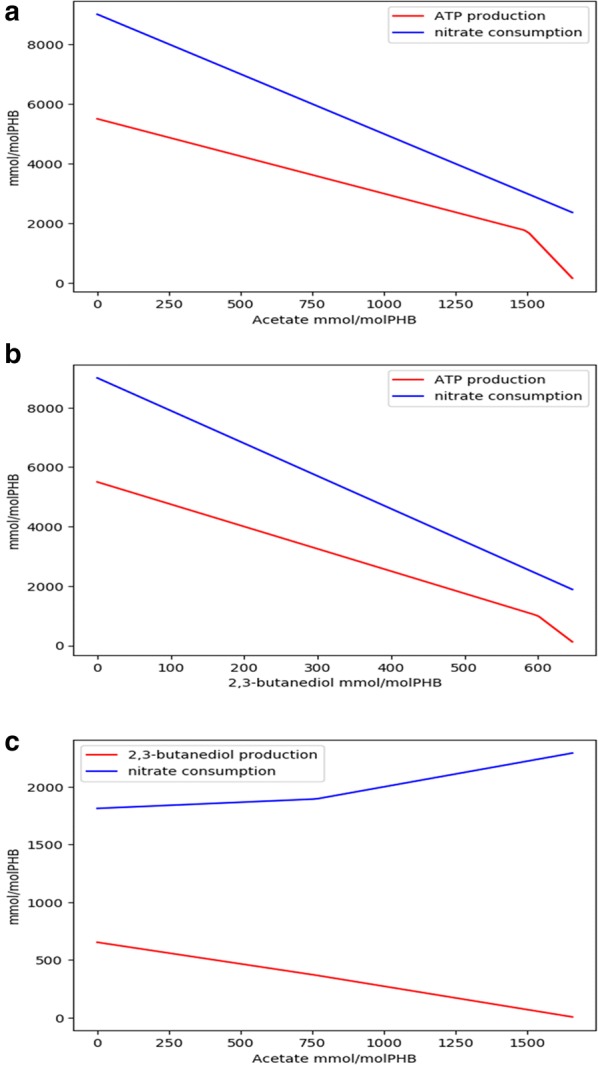
Predicted maximal ATP produced and nitrate consumed per mol of degraded PHB versus secreted acetate (**a**). Predicted maximal ATP produced and nitrate consumed per mol of degraded PHB versus secreted butane-2,3-diol (**b**). Predicted maximal butane-2,3-diol produced and nitrate consumed per mol of degraded PHB versus secreted acetate (**c**)

It has been reported that *M. parvus*, in the absence of oxygen, can secrete acetate and butane-2,3-diol [14]. This phenomenon was attributed to the fermentation of stored PHB able to supply ATP for maintenance. The reconstructed GSMM was used to assess the metabolic feasibility of PHB fermentation by setting to zero the oxygen uptake rate and maximizing ATP production from PHB. Under these conditions, ATP production was coupled to denitrification. If nitrate consumption was set to zero, no ATP production was predicted by the model. Therefore, PHB is likely to be used by *M. parvus* cells as an energy source under anoxic conditions only if nitrate is present as electron acceptor. Production of acetate and butane-2,3-diol from PHB, using nitrate as electron acceptor, was also modeled. The simulations were carried out by setting the PHB consumption to 1 mol and optimizing ATP production for different productions of acetate and butane-2,3-diol. Maximal ATP production corresponded to zero production of acetate and butane-2,3-diol. Thus, the optimal production of any of these two compounds appeared to be incompatible with ATP production. Figure 4 shows Pareto plots depicting the trade-off between ATP production and acetate and butane-2,3-diol production, and the nitrate consumption required in each case. Therefore, model predictions suggested that the production of the mentioned compounds was likely to be an overflow phenomenon due to the fact that PHB degradation rate is faster than its oxidation by nitrate, and the non-oxidized carbon is secreted in the form of the mentioned compounds. Figure 4 shows that less nitrate is necessary for acetate or butane-2,3-diol production than for ATP production (which involves a complete oxidation of PHB to CO_2_).

### Comparative analysis of the methane monooxygenase complexes of *Methylocystis parvus*

Methane oxidation is likely to be the step limiting the growth rate of methanotrophs. The reduced duplication time observed in *M. parvus* OBBP during PHB and methane co-consumption (which was accompanied by a slightly lower specific methane degradation rate), seems to confirm this hypothesis. Other evidence suggesting that methane oxidation is typically a limiting step was provided by knockout experiments in *M.* sp. SC2 [6]. *M. parvus* has a specific growth rate at 30 °C of 0.107 h^−1^ [7], while *M.* sp. SC2 was reported to have a specific growth rate of 0.074 h^−1^ [6] and *M. hirsuta* has a specific growth rate of only 0.048 h^−1^ [17]. Therefore, it is interesting to compare the pMMO genes of these organisms in order to infer possible reasons for the observed differences in specific growth rates.

**Fig. 5:**
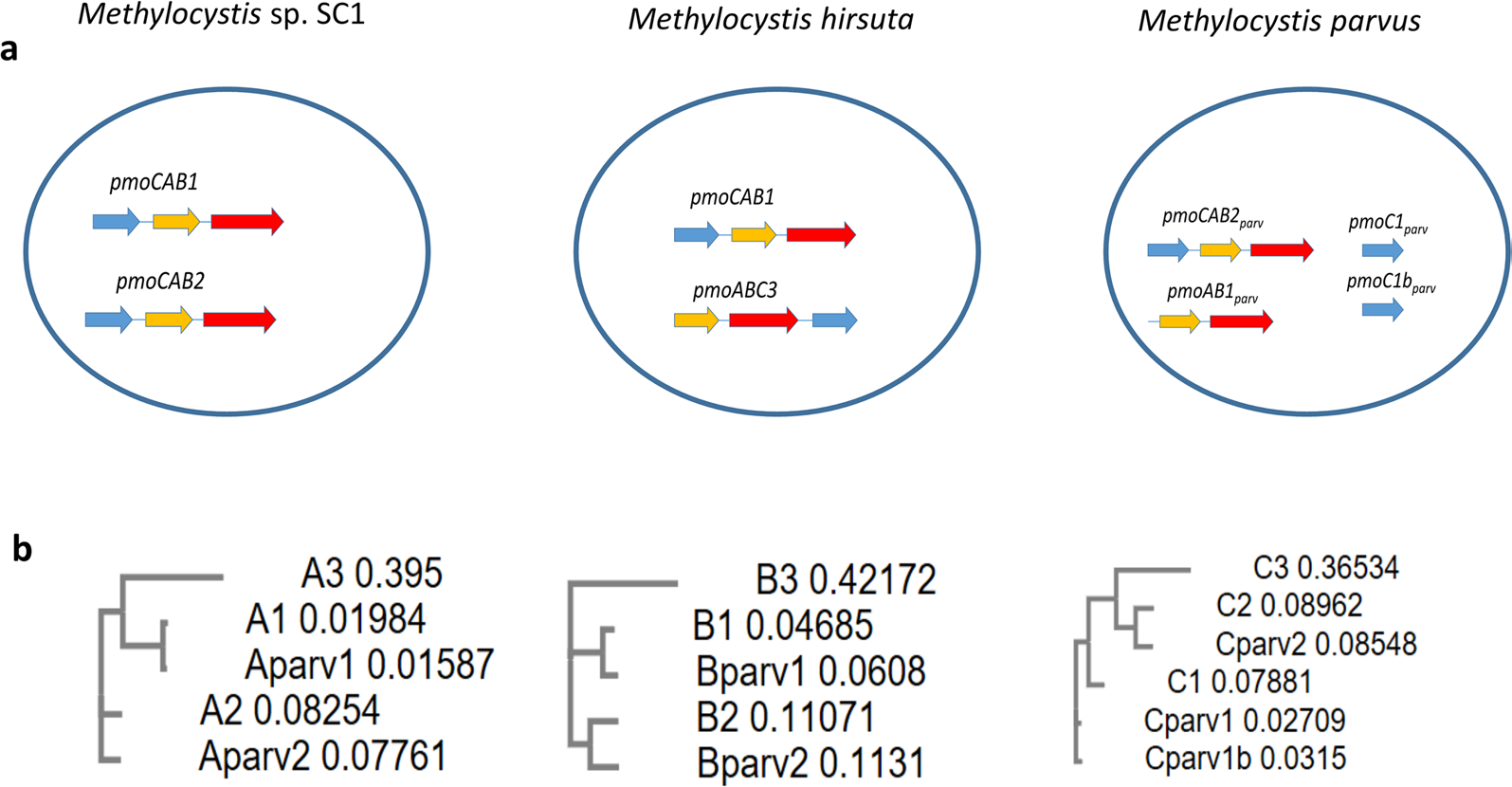
Structure of the gene clusters of particulate methane monooxygenases present in *M. parvus*, *M. hirsuta* and *M.* sp. SC2 (**a**). Phylogenetic trees showing the relative sequence similarity of each of the three subunits (**b**)

**Fig. 6:**
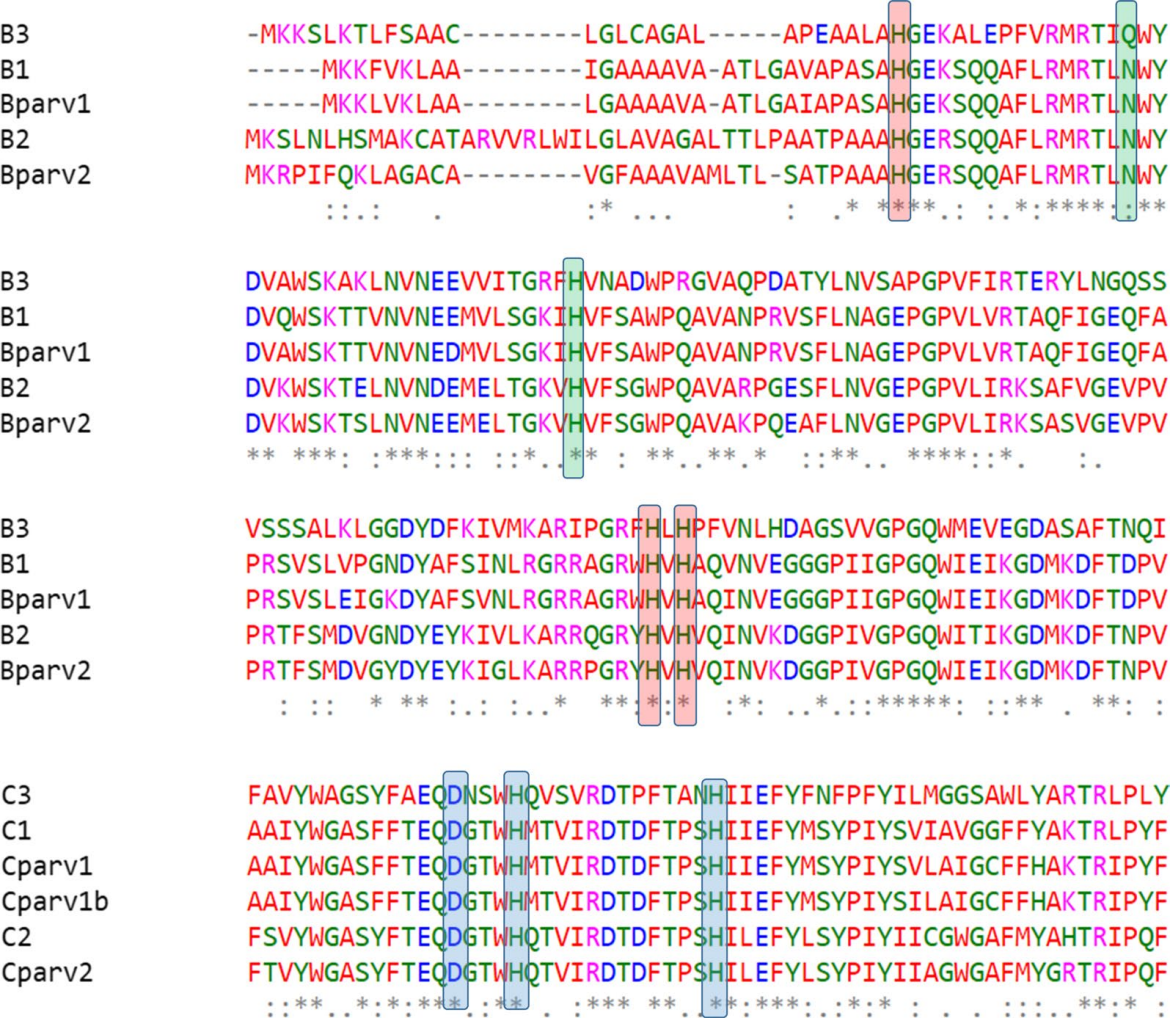
Multiple alignments showing the metallic catalytic sites of pMMO. The dinuclear copper site formed by three histidine residues in subunit B is highlighted in red. The zinc site, formed by two histidine and one aspartic acid residue in subunit C, is highlighted in blue. The mononuclear cooper site, formed by a histidine and an asparagine residue in subunit B, is highlighted in green. In the subunit B3, present in *M. hirsuta*, asparagine was changed into glutathione

Particulate methane monooxygenases have a trimeric architecture, consisting of three copies each of the pmoA, pmoB and pmoC sub-units [19]. These subunits are normally arranged forming gene clusters. *M.* sp. SC2 possesses two different gene clusters referred as pmoCAB1 and pmoCAB2 (the order of letters reflects the order in which the genes of the three different subunits are arranged), of which pmoCAB2 expression appears to be associated to higher affinity for methane at low concentrations [6]. *M. hirsuta* has a pmoCAB1 cluster identical to the one present in *M.* sp. SC2 and an alternative cluster named pmoABC3. The cluster pmoCAB2 is absent in *M. hirsuta*. In contrast, *M. parvus* OBBP has a gene cluster closely related but not identical to the pmoCAB2 cluster from *M.* sp. SC2. This gene cluster was herein referred to as pmoCAB2_parv_. A second cluster with only 2 subunits (A and B), which are similar (but not identical) to the A and B subunits of the cluster pmoCAB1, was found in *M. parvus*. This cluster was referred to as pmoAB1_parv_. Finally, two separate C sub-units, very similar to each other and similar to the C sub-unit of pmoCAB1, are found in the genome of *M. parvus*. These enzymes were herein referred to as pmoC1_parv_ and pmoC1b_parv_, respectively. Figure 5 summarizes the structure of the pmo genes in each of the three considered strains and provides phylogenetic trees showing the similarity among all the mentioned pmo subunits. Multiple alignments of the complete protein sequences are provided as Additional file 1 (Fig. 6 shows alignments of the regions in which the catalytic sites are located).

Multiple alignments and phylogenetic trees comparing the three pMMO subunits were carried out using the software MUSCLE [20]. The performed alignments revealed that the dinuclear copper site (subunit C) and the zinc site (subunit B), characteristic of pMMO [19], were well conserved, while the mononuclear copper site (subunit B) is conserved in all the cases except in the pmoABC3 cluster, characteristic of *M. hirsuta*. In this case, asparagine is substituted by glutathione. Multiple alignments of the catalytic sites are presented in Fig. 6.

## Discussion

Based on the sequenced genome of *M. parvus* OBBP, the first functional GSMM for this organism was herein reconstructed. The model was able to accurately predict biomass yields on methane (under the assumption of methane oxidation using the “redox arm” mechanism). The model was equally able to predict accurately both oxygen consumption and biomass production in the case of simultaneous co-consumption of methane and stored PHB. The metabolic flux distribution predicted by the model revealed that the stored PHB is used during its co-consumption with methane to replenish the serine cycle with glyoxylate and the TCA cycle with succinyl-CoA. This allows anabolic precursors to be withdrawn from these cycles leading to higher specific growth rates (reduced cell duplication times). This anaplerotic function of the stored PHB as a source of the glyoxylate replenishing the serine cycle, had been previously suggested [21]. However, other authors [7] observed that glyoxylate addition to the growth medium did not decrease PHB consumption and concluded that PHB did not mediate glyoxylate consumption. The glyoxylate supplied to the growth medium in the previously mentioned experiment, was indeed not consumed by the cells. This fact and the model predictions suggested that external glyoxylate is just not being uptaken by the cells, which keep on relying on PHB degradation. This hypothesis was also consistent with the fact that no specific glyoxylate transporters were annotated in the genome of *M. parvus*. In contrast, formate supplied to the growth medium was uptaken by the cells [7], which was consistent with the presence of a formate transporter (with identifier peg.2893), leading to a lower PHB consumption rate. Formate is transformed into methylene tetrahydrofolate and enters the serine cycle in the same way as all other C1 compounds, which ultimately increases the rate of the serine cycle. Based on the fact that malyl-CoA lyase catalyzes both a step of the serine cycle and the breakdown of l-erytro-3-methylmalyl-CoA (originated from PHB) into glyoxylate and propionyl-CoA, it is reasonable that an increased flux of the serine cycle, caused by the uptake of formate, will result in a lower rate of PHB degradation, as both pathways are competing for the same enzyme.

The model also revealed that denitrification was the only mechanism for the stored PHB to be used as energy source in the absence of oxygen. Therefore, the anaerobic fermentation of PHB, previously hypothesized in literature [14], was not supported by the model. This hypothesis was based on the observation of secreted acetate and butane-2,3-diol by *M. parvus* under anoxic conditions. The model revealed that the production of these compounds from stored PHB also requires denitrification. However, a lower nitrate consumption is required for the secretion of these compounds than for the complete oxidation of PHB. The observed production of acetate and butane-2,3-diol was therefore more likely due to an overflow mechanism caused by the faster PHB utilization compared to the denitrification rate required for full oxidation of PHB to CO_2_.

The yield on methane of *M. parvus* is the same as the yields (experimental and predicted by their GSMMs) of *M. hirsuta* and *Methylocystis* sp. SC2. However, *M. parvus* exhibited a higher specific growth rate, and thus a higher specific methane oxidation rate, than the two other species. *M. parvus* has a similar system of pMMO enzymes than that of *M.* sp. SC2. In particular, the metallic catalytic sites are well conserved in both organisms and no conclusive statements can be made regarding the higher specific oxidation rates of *M. parvus* compared to *M.* sp. SC2. *M. hirsuta* is characterized by a different pmoABC3 gene cluster (absent in the two other strains) in which the mononuclear copper site present in the B subunit of pMMO is mutated. The gene cluster pmoCAB1 is present in both *M. hirsuta* and *M.* sp. SC2. Closely related enzymes to those in the pmoCAB1 cluster are also present in *M. parvus*, but arranged in a pmoAB1_parv_ cluster plus two independent pmoC1 genes. The absence of the pmoCAB2 cluster in *M. hirsuta* might be related to its low specific methane oxidation rate. After removing the pmoCAB2 cluster from *M.* sp. SC2 [6], this strain showed decreased specific growth rates similar to those observed in *M. hirsuta*, which suggested that the presence of the pmoABC3 cluster does not compensate the lack of the pmoCAB2 cluster in this strain.

In summary, the GSMM presented in this article provided accurate quantitative predictions of *M. parvus* OBBP metabolism. Its high PHB accumulation capacity and high specific methane oxidation rate makes *M. parvus* OBBP a promising cell factory, while the availability of a predictive GSMM for this organism will foster future developments as a metabolic engineering platform.

## Materials and methods

### Reconstruction of Genome Scale Metabolic Models

The genome of *M. parvus* OBBP [10] has been obtained from GeneBank (Accession Number AJTV00000000). The genome was annotated with RAST [16]. The resulting annotation, in Excel format, has been made available at https://github.com/SergioBordel/ModelsMethanotrophs. A draft metabolic model was obtained from the genome annotation using SEED [15]. The resulting draft was manually curated following three steps: first of all, each reaction gene association was checked manually comparing the gene annotation with its associated reaction in the draft. Wrong associations were corrected manually. For instance, the reaction corresponding to nitrate reduction, with identifier rxn10121_c0, was associated in the draft model with the gene peg.4531, which was annotated by RAST as nitrite reductase large subunit (EC. 1.7.1.4). The association was changed manually to nitrate reductases (EC. 1.4.99.4), of which four different iso-enzymes were found in the genome (peg.4533, peg.4534, peg.1308, peg.4022). Secondly, the complete genome annotation was manually checked in order to identify genes coding metabolic enzymes that had not been added to the model. For instance, a intracellular PHB depolymerase, which catalyzes PHB hydrolysis, was found in the genome with identifier peg.911 but was absent from the draft model. Finally, reaction directionality was manually curated in order to avoid the possibility of thermodynamically unfeasible ATP production or extrusion of protons through the membrane against its concentration gradient, without energy expenditure. The resulting model was deposited in BioModels [22] under the Accession Number MODEL1904120001.

### Simulations of metabolic fluxes

All model manipulations (addition and deletion of reactions, changes in gene associations and reaction directionality etc.), as well as simulations using flux balance analysis (FBA), were carried out using the python library COBRApy [22].

### Strain, chemicals and culture conditions

The strain *M. parvus* OBBP was obtained from Biopolis S.L. (Valencia, Spain). The bacterium was cultured in Whittenbury nitrate mineral medium (pH 6.8) [23]. Cultures were carried out in 120 mL serum bottles crimp sealed under sterile conditions containing 20 mL of mineral medium. The bottle headspaces were flushed with pure oxygen and different volumes of oxygen were extracted and replaced with pure methane under sterile conditions. Leading to initial methane headspace concentrations of 19, 29, 40, 59 and 74 mgCH_4_ L^−1^. Cultures were carried out in an orbital shaker at 200 rpm and at 30 °C.

### Analytical methods

Gas concentrations of CH_4_, O_2_ and CO_2_ in the headspace of the serum bottles were determined using gas chromatography in a Bruker 430 GC-TCD (Bruker, Palo Alto, USA) with two columns: a CP-Molsieve 5A (15 m × 0.53 mm × 10 mm) [24] and a CP-PoraBOND Q (25 m × 0.53 mm × 10 mm). Biomass concentration was determined using culture absorbance measurements at 600 nm (OD600), which was previously correlated with total suspended solids (TSS) as described previously [25].

## Additional file

**Additional file 1.** Multiple protein alignments of each of the three pMMO subunits.

## Data Availability

All the data have been made available at: https://github.com/SergioBordel/ModelsMethanotrophs.

## References

[1] Abbasi T, Tauseef SM, Abbasi SA (2012). Anaerobic digestion for global warming control and energy generation, an overview. Renew Sustain Energy Rev.

[2] Strong PJ, Kalyuzhnaya M, Silverman J, Clarke WP (2016). A methanotroph-based biorefinery: potential scenarios for generating multiple products from a single fermentation. Bioresour Technol.

[3] Comer AD, Long MR, Reed JL, Brian FP (2017). Flux balance analysis indicates that methane is the lowest cost feedstock for microbial cell factories. Metab Eng Commun.

[4] de la Torre A, Metivier A, Chu F, Laurens LML, Beck DAC, Pienkos PT, Lindstrom ME, Kaluzhnaya MG (2015). Genome-scale metabolic reconstruction and theoretical investigation of methane conversión in *Methylomicrobium buryatense* strain 5G(B1). Microb Cell Fact.

[5] Akberdin IR, Thompson M, Hamilton R, Desai N, Alexander D, Henard CA, Guarnieri MT, Kalyuzhnaya MG (2018). Methane utilization in *Methylomicrobium alcaliphilum* 20ZR: a systems approach. Sci Rep.

[6] Baani M, Liesack W (2008). Two isozymes of particulate methane monooxygenase with different methane oxidation kinetics are found in *Methylocystis* sp. strain SC2. Proc Natl Acad Sci USA.

[7] Pieja AJ, Sundstrom ER, Criddle CS (2011). Poly-3-hydroxybutyrate metabolism in the type II methanotroph *Methylocystis parvus* OBBP. Appl Environ Microbiol.

[8] García-Pérez T, López JC, Passos F, Lebrero R, Revah S, Muñoz R (2018). Simultaneous methane abatement and PHB production by *Methylocystis hirsuta* in a novel gas-recycling bubble column bioreactor. Chem Eng J.

[9] Handrick R, Reinhardt S, Jendrossek D (2000). Mobilization of poly(3-hydroxybutirate) in *Ralstonia eutrophia*. J Bacteriol.

[10] Del Cerro C, García JM, Rojas A, Tortajada M, Ramón D, Galán B, Prieto MA, García JL (2012). Genome sequence of the methanotrophic poly-β-hydroxybutyrate producer *Methylocystis* parvus OBBP. J Bacteriol.

[11] Bordel S, Rodríguez E, Muñoz R (2018). Genome sequence of *Methylocystis hirsuta* CSC1, a polyhydroxyalkanoate producing methanotroph. MicrobiologyOpen.

[12] Dam B, Dam S, Blom J, Liesack W (2012). Genome analysis coupled with physiological studies reveals a diverse nitrogen metabolism in *Methylocystis* sp. strain SC2. PLoS ONE.

[13] Kalyuzhnaya MG, Puri AW, Lidstrom ME (2015). Metabolic engineering in methanotrophic bacteria. Metab Eng.

[14] Vecherskaya M, Dijkema C, Ramírez Saad H, Stams AJM (2009). Microaerobic and anaerobic metabolism of a *Methylocystis parvus* strain isolated from a denitrifying bioreactor. Environ Microbiol Rep.

[15] Overbeek R, Olson R, Pusch GD, Olsen GJ, Davis JJ, Disz T, Edwards RA, Gerdes S, Parrello B, Shukla M, Vonstein V, Wattam AR, Xia F, Stevens R (2014). The SEED and the rapid annotation of microbial genomes using subsystems technology (RAST). Nucleic Acids Res.

[16] Overbeek R, Begley T, Butler RM, Choudhuri JV, Chuang HY, Cohoon M, de Crécy-Lagard V, Diaz N, Disz T, Edwards R, Fonstein M, Frank ED, Gerdes S, Glass EM, Goesmann A, Hanson A, Iwata-Reuyl D, Jensen R, Jamshidi N, Krause L, Kubal M, Larsen N, Linke B, McHardy AC, Meyer F, Neuweger H, Olsen G, Olson R, Osterman A, Pornov V, Pusch GD, Rodionov DA, Rückert C, Steiner J, Stevens R, Thiele I, Vassieva O, Ye Y, Zagnitko O, Vonstein V (2005). The subsystems approach to genome annotation and its use in the project to annotate 1000 genomes. Nucleic Acids Res.

[17] Bordel S, Rodíguez Y, Hakobyan A, Rodríguez E, Lebrero R, Muñoz R (2019). Genome scale metabolic modeling reveals the metabolic potential of three Type II methanotrophs of the genus *Methylocystis*. Metab Eng.

[18] Becker SA, Price ND, Palsson BO (2006). Metabolite coupling in genome-scale metabolic networks. BMC Bioinform.

[19] Culpepper MA, Rosenzweig AC (2012). Architecture and active site of particulate methane monooxygenase. Crit Rev Biochem Mol Biol.

[20] Edgard RC (2004). MUSCLE: multiple sequence alignment with high accuracy and high throughput. Nucelic Acids Res.

[21] Korotkova N, Lindstrom ME (2001). Connection between poly-beta-hydroxybutyrate biosynthesis and growth on C(1) and C(2) compounds in the methylotroph *Methylobacterium extorquens* AM1. J Bacteriol.

[22] Ebrahim A, Lerman JA, Palsson BO, Hyduke DR (2013). COBRApy: constraints-based reconstruction and analysis for python. BMC Syst Biol.

[23] Whittenbury R, Phillips KC, Wilkinson JF (1970). Enrichment, isolation and some properties of methane-utilizing bacteria. J Gen Microbiol.

[24] López JC, Arnáiz E, Merchán L, Lebrero R, Muñoz R (2018). Biogas-based polyhydroxyalkanoates production by *Methylocystis hirsuta*: a step further in anaerobic digestión biorefineries. Chem Eng J.

[25] López JC, Quijano G, Pérez R, Muñoz R (2014). Assessing the influence of CH4 concentration during culture enrichment on the biodegradation kinetics and population structure. J Environ Manag.

