# Supplementary figures and images for: Reconstruction of a Genome Scale Metabolic Model of the polyhydroxybutyrate producing methanotroph Methylocystis parvus OBBP
Source: Microb Cell Fact. 2019 Jun 7;18:104. doi: 10.1186/s12934-019-1154-5 (PMC6554988; doi:10.1186/s12934-019-1154-5)

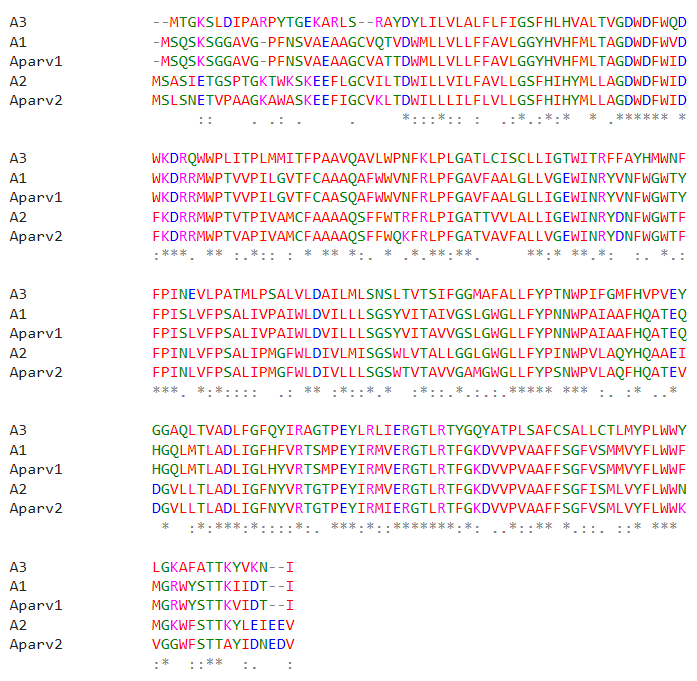


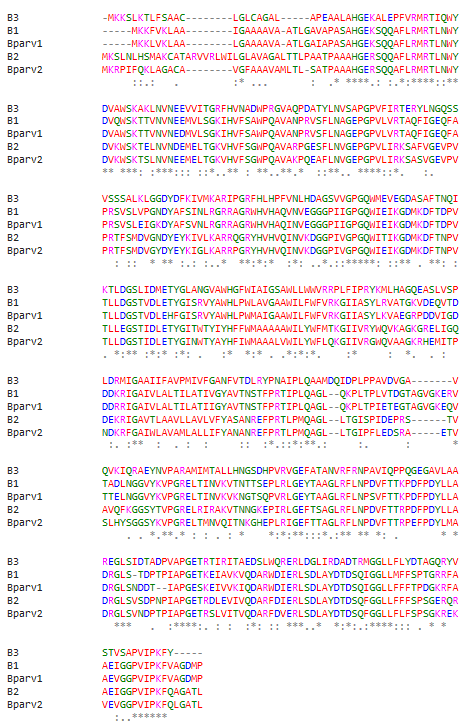


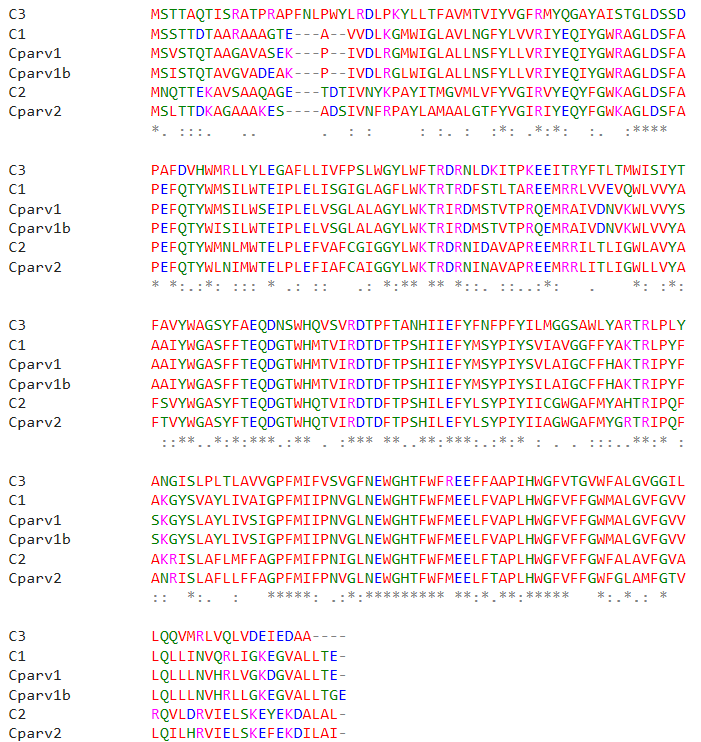

Supplement: Supplementary file 1 — Additional file 1. Multiple protein alignments of each of the three pMMO subunits. [file 12934_2019_1154_MOESM1_ESM.docx]
